# Three new species of *Thelepus* Leuckart, 1849 from Europe and a re-description of *T. cincinnatus* (Fabricius, 1780) (Annelida, Terebellidae)

**DOI:** 10.3897/zookeys.759.22981

**Published:** 2018-05-17

**Authors:** Igor Jirkov

**Affiliations:** 1 Department of Hydrobiology, Biological Faculty, Moscow Lomonosov State University, Moscow, Russia

**Keywords:** cosmopolitan species, generic characteristic, identification key, morphological characters, Polychaeta, taxonomic revision, taxonomy

## Abstract

The review of a large amount of material previously identified as the terebellid annelid, *Thelepus cincinnatus* (Fabricius, 1780) shows that, within European waters from the Mediterranean to the North Pole, this species should be split into four species, three of which (*T. davehalli* **sp. n.**, *T. marthae* **sp. n.**, and *T. parapari* **sp. n.**) are newly described here and *T. cincinnatus* s. str. is re-described. These four species each show distinct distribution ranges. *Thelepus cincinnatus* has notopodia on almost all segments and numerous eyespots; it inhabits the high boreal and arctic shelf and the North Atlantic slope, and probably also occurs on the North Pacific shelf and slope. *Thelepus marthae* **sp. n.** has no eyespots and inhabits deep waters of the high Arctic. *Thelepus davehalli* **sp. n.** has no eyespots and has notopodia on 1/2 to 2/3 of the anterior of the body; it inhabits boreal shelf waters (from Iceland to the Mediterranean) below the tidal front. *Thelepus parapari* **sp. n.** differs from the previous three species in that the uncini of the first uncinigerous segment has two teeth above the main fang; it inhabits shallow, coastal waters of the Mediterranean, inshore from the tidal front.

## Introduction

Thirty years ago, Hutchings and Glasby stated “Many species of *Thelepus* have been described, but many inadequately, and type material in most cases needs to be re-examined” (Hutchings and Glasby 1987: 226). Unfortunately, this situation has persisted and led to the appearance of another “cosmopolitan species”, which is, in reality, a complex of pseudocryptic species. The type species of *Thelepus*, *T. cincinnatus*, was reported as cosmopolitan by Hartmann-Schröder (1996). Despite being absent from the tropics (thus not truly cosmopolitan), its reported range is very wide: from the eastern North Atlantic, from Cape Verde in the south, through the Mediterranean to the western North Atlantic and the Caribbean, the North Polar Basin and the North Pacific: Japan and Washington (Uschakov 1955; Imajima and Hartman 1964; Hobson and Banse 1981; Holthe 1986; Jirkov 2001). Hartman (1966) also reported *T. cincinnatus* amongst Antarctic polychaetes. Existing records indicate a vertical distribution from the eulittoral zone to a depth of ca. 4000 m (Holthe 1986). However, recent investigations indicate the species’ true range is not as extensive, with the Caribbean for example already excluded from its range (Londoño-Mesa 2009). The extensive range and habitat preferences of *T. cincinnatus* were investigated during the examination of European material for the Fauna Ibérica Project. As a result, instead of the single species *T. cincinnatus*, these records indicate four species: *T. cincinnatus* s. str. and three new species described here. The previously reported range of *T. cincinnatus* within the Arctic and North Atlantic is thus divided between these four species. It should be noted that *T. cincinnatus* also has a long list of subjective synonyms: 12 according to Bellan (2008); Fauvel (1927) also accepted *T. nucleolata* (valid according to Hsueh and Li 2016) as a synonym of *T. cincinnatus*. Unfortunately, all these synonymized taxa were described at least a century ago, and have poor original descriptions and an absence of type material; it is not possible to confirm their taxonomic status.

## Materials and methods

**Figure 1.**
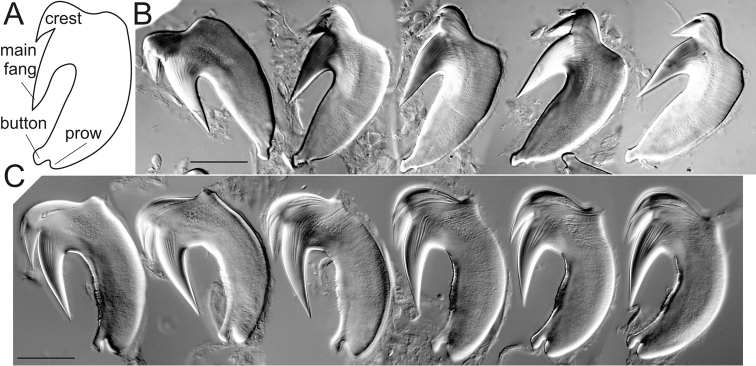
Uncinial parts and uncini of *T. triserialis* and *T. setosus***. A** uncinial parts according to Nogueira et al. (2010) **B** uncini U1 *T. setosus* APEM 413169 **C** uncini U1 *T. triserialis* MNCN 2508. Scale bars: 20 μm.

The higher taxonomy used in this paper follows Read and Fauchald (2018). Morphological terms used in this paper follow Nogueira et al. (2010) and are explained in Fig. 1A. Taxonomic abbreviations used are as follows:

**BS** branchiferous segment;

**C** chaetiger;

**S** segment;

**T** thoracic;

**U** unciniger.

The number following the abbreviation refers to the number of the segment (e.g. BS1 means branchiferous segment 1). Institutional abbreviations used are as follows:

**APEM** APEM Ltd., UK;

**Aveiro** Biology Department of the Universidade de Aveiro, Portugal;

**KGB** Department of Hydrobiology Moscow Lomonosov State University, Russia;

**MNCN** Museo Nacional de Ciencias Naturales, Madrid, Spain;

**ZIN** Zoological Institute of Russian Academy of Science, St. Petersburg, Russia.

This study was based almost exclusively on collections from the KGB. Mediterranean specimens were examined from the collection at the MNCN; specimens from UK waters were examined from the APEM collection. All material, if not stated otherwise, is deposited at KGB. Sampling data are given in Table 1.

**Table 1.** Collection data and institutional repository for all investigated material. Abbreviations (in addition to those in Material & methods): SP-22 – drifting ice station Severniy Polus 22; VNIRO – Russian Federal Research Institute of Fisheries and Oceanography, n/a – not applied, - – no data.

Species	Ship or expedition	Cruise. station or collection number	Latitude	Longitude	Depth, m	T °C	S‰	Day	Month	Year	Number of specimens	Collection
*T. cincinnatus*	Alaid	30.6	74°14'N, 19°20'E		64	0.91	34.8	1	7	1980	73	KGB
*T. cincinnatus*	Alaid	30.7	74°30'N, 28°00'E		388	0.86	34.9	4	7	1980	1	KGB
*T. cincinnatus*	Alaid	30.8	74°30'N, 32°30'E		190	-1.53	34.8	4	7	1980	136	KGB
*T. cincinnatus*	Alaid	30.13	68°51'N, 37°20'E		75	2.68	34.0	11	7	1980	15	KGB
*T. cincinnatus*	Molchanov	14.9303	69°53'N, 41°39'E		95	-	-	18	4	1986	2	KGB
*T. cincinnatus*	Otkupshikov	181.19	69°15'N, 35°48'E		145	1.70	34.6	9	7	1978	7	KGB
*T. cincinnatus*	Persey	5.183	76°33'N, 41°12'E		230	-	-	1	9	1924	5	KGB
*T. cincinnatus*	Persey	5.220	75°09'N, 18°47'E		35	-	-	30	9	1924	1	KGB
*T. cincinnatus*	Persey	12.650	74°36'N, 32°34'E		172	-1.35	-	7	6	1927	11	KGB
*T. cincinnatus*	Persey-3	14.3024	47°20'N, 49°00'W		115	-	-	8	9	1975	3	KGB
*T. cincinnatus*	Persey-3	15.3341	44°43'N, 49°02'W		217	-	-	6	5	1976	1	KGB
*T. cincinnatus*	Persey-3	15.3521	48°20'N, 52°06'W		185	-1.00	-	19	6	1976	2	KGB
*T. cincinnatus*	Persey-3	15.3529	47°58'N, 49°45'W		185	-	-	20	6	1976	20	KGB
*T. cincinnatus*	Persey-3	14.2724	47°00'N, 47°30'W		215	2.00	-	25	6	1975	3	KGB
*T. cincinnatus*	Saratov	16.1374	69°10'N, 36°00'E		47	7.04	34.0	26	8	1947	2	KGB
*T. cincinnatus*	Sevastopol	5.1078	63°55'N, 13°03'W		650	3.38	35.0	15	7	1957	5	KGB
*T. cincinnatus*	Sevastopol	9.1580	62°00'N, 24°56'W		1350	-	-	26	6	1958	10	KGB
*T. cincinnatus*	Sevastopol	10.1768	65°47'N, 11°02'W		173	2.86	34.9	16	10	1958	1	KGB
*T. cincinnatus*	Sevastopol	8.1427	64°45'N, 12°31'W		157	1.34	34.8	9	4	1958	26	KGB
*T. cincinnatus*	Sevastopol	8.1411	61°50'N, 1°45'E		185	7.37	35.3	6	4	1958	4	KGB
*T. cincinnatus*	Shmidt	26.2301	79°20'N, 45°53'E		65	-	-	11	8	1986	2	KGB
*T. cincinnatus*	Tunetz	105.22	74°30'N, 20°10'E		94	-0.12	34.7	7	7	1978	45	KGB
*T. cincinnatus*	Tunetz	105.23	74°30'N, 31°20'E		245	-0.76	34.9	8	7	1978	3	KGB
*T. cincinnatus*	VNIRO	2003.223808	67°07'N, 41°24'E		20	-	-	-	-	2003	18	KGB
*T. cincinnatus*	VNIRO	2003.223820	68°08'N, 39°46'E		8	-	-	-	-	2003	2	KGB
*T. cincinnatus*	VNIRO	2003.223900	69°29'N, 32°39'E		12	-	-	-	-	2003	2	KGB
*T. cincinnatus*	VNIRO	2003.223984	69°28'N, 32°35'E		9	-	-	-	-	2003	2	KGB
*T. cincinnatus*	VNIRO	2003.2231011	67°14'N, 41°16'E		15	-	-	-	-	2003	2	KGB
*T. cincinnatus*	n/a	129/33188	75°05'N, 113°25'E		36	-	-	27	8	1912	2	ZIN
*T. cincinnatus*	n/a	132/33190	77°21'N, 107°E		37	-1.2	-	26	8	1913	3	ZIN
*T. cincinnatus*	n/a	139/33197	75°17'N, 113°50'E		43	1.5	-	9	9	1912	1	ZIN
*T. cincinnatus*	n/a	208/33265	71°08'N, 175°52'W		41	-	-	7	9	1929	1	ZIN
*T. cincinnatus*	n/a	212/33268	80°47'N, 89°50'E		52	-	-	31	8	1930	2	ZIN
*T. davehalli*	Sevastopol	5.1089	62°30'N, 7°48'W		150	8.78	35.2	16	7	1957	2	KGB
*T. davehalli*	Sevastopol	5.1091	61°59'N, 6°07'W		130	8.73	35.3	17	7	1957	5	KGB
*T. davehalli*	Sevastopol	5.1102	60°35'N, 0°36'W		135	9.24	35.4	18	7	1957	36	KGB
*T. davehalli*	Sevastopol	5.1104	60°35'N, 0°45'E		130	8.16	35.4	18	7	1957	6	KGB
*T. davehalli*	Sevastopol	5.1105	60°35'N, 1°21'E		130	7.72	35.4	19	7	1957	1	KGB
*T. davehalli*	Sevastopol	5.1157	64°56'N, 24°25'W		155	8.17	35.1	31	7	1957	1	KGB
*T. davehalli*	Sevastopol	8.1443	62°30'N, 7°12'W		94	6.38	35.3	11	4	1958	1	KGB
*T. davehalli*	Sevastopol	8.1453	62°00'N, 6°14'W		112	6.34	35.2	16	4	1958	207	KGB, MNCN
*T. davehalli*	Sevastopol	8.1464	60°34'N, 0°36'W		145	6.2	35.3	17	4	1958	25	KGB
*T. davehalli*	Sevastopol	8.1465	60°35'N, 0°01'W		110	6.18	35.3	17	4	1958	1	KGB
*T. davehalli*	Sevastopol	8.1466	60°35'N, 0°47'E		128	5.89	35.4	18	4	1958	8	KGB
*T. davehalli*	Sevastopol	8.1468	60°35'N, 2°05'E		127	5.91	35.3	18	4	1958	1	KGB
*T. davehalli*	Sevastopol	8.1490	73°40'N, 20°34'E		495	1.73	35.1	28	4	1958	1	KGB
*T. davehalli*	Sevastopol	10.1790	62°32'N, 7°00'W		100	9.42	35.2	22	10	1958	2	KGB
*T. davehalli*	Sevastopol	10.1792	62°00'N, 6°14'W		115	9.47	35.1	24	10	1958	32	KGB
*T. davehalli*	Sevastopol	10.1801	60°56'N, 1°34'W		134	10.3	35.4	25	10	1958	1	KGB
*T. davehalli*	Sevastopol	10.1803	60°35'N, 0°34'W		140	11.3	35.3	25	10	1958	16	KGB
*T. davehalli*	Sevastopol	10.1804	60°35'N, 0°02'E		110	11.1	35.3	26	10	1958	4	KGB
*T. davehalli*	Sevastopol	10.1805	60°35'N, 0°35'E		138	9.18	35.4	26	10	1958	13	KGB
*T. davehalli*	Sevastopol	15.2548	62°30'N, 7°15'W		95	9.01	35.2	28	11	1959	15	KGB
*T. davehalli*	Sevastopol	15.2574	60°36'N, 0°45'E		130	9.22	35.3	10	12	1959	2	KGB
*T. davehalli*	Sevastopol	15.2587	62°00'N, 6°12'W		120	8.4	35.2	12	12	1959	43	KGB
*T. davehalli*	n/a	DBUA0000389.01.	40°30'–40°50'	8°40'–9°30'W	101	-	-		7–8	1994	2	Aveiro
*T. davehalli*	n/a	APEM ADDGT09	55°49'N, 0°07'E		79	-	-	17	3	2014	2	KGB
*T. davehalli*	n/a	16.01/488	Naples		-	-	-	-	-	-	3	MNCN
*T. marthae*	Sevastopol	15.2512	65°45'N, 5°00'E		1000	-0.64	34.9	17	11	1959	6	KGB
*T. marthae*	Alaid	30.3	68°00'N, 10°00'E		958	-0.79	34.9	13	6	1980	501	KGB, MNCN
*T. marthae*	Persey	14.860	73°33'N, 59°53'E		380	-1.64	-	21	9	1927	2	KGB
*T. marthae*	Sevastopol	5.1068	65°45'N, 8°01'W		1230	-0.9	-	12	7	1957	2	KGB
*T. marthae*	Sevastopol	5.1097	61°21'N, 3°12'W		1275	-0.9	34.9	18	7	1957	6	KGB
*T. marthae*	Sevastopol	5.1114	63°00'N, 4°27'E		860	2.8	34.9	20	7	1957	1	KGB
*T. marthae*	Sevastopol	5.1212	67°39'N, 22°36'W		650	-0.47	35.0	7	8	1957	44	KGB
*T. marthae*	Sevastopol	5.1214	67°54'N, 21°57'W		805	-0.52	34.9	7	8	1957	1	KGB
*T. marthae*	Sevastopol	5.1216	67°53'N, 20°15'W		970	-0.32	35.0	7	8	1957	2	KGB
*T. marthae*	Sevastopol	8.1358	66°27'N, 6°02'E		770	-0.89	34.9	25	3	1958	2	KGB
*T. marthae*	Sevastopol	8.1372	69°40'N, 8°00'W		960	-0.88	34.9	29	3	1958	1	KGB
*T. marthae*	Sevastopol	8.1383	66°30'N, 12°40'W		920	-0.9	34.9	31	3	1958	2	KGB
*T. marthae*	Sevastopol	10.1702	66°38'N, 4°59'E		1125	-0.94	34.9	27	9	1958	2	KGB
*T. marthae*	Sevastopol	10.1723	68°34'N, 14°04'W		1280	-0.81	34.9	2	10	1958	2	KGB
*T. marthae*	Sevastopol	10.1758	62°44'N, 2°41'W		890	-0.64	34.9	13	10	1958	1	KGB
*T. marthae*	Sevastopol	10.1770	67°18'N, 23°33'W		511	-0.4	35.0	17	10	1958	47	KGB
*T. marthae*	Sevastopol	10.1772	67°49'N, 24°40'W		1510	-0.74	35.1	18	10	1958	13	KGB
*T. marthae*	Sevastopol	15.2457	71°06'N, 10°21'W		1360	-0.79	34.9	6	11	1959	4	KGB
*T. marthae*	Sevastopol	15.2549	63°00'N, 7°30'W		710	-0.2	34.9	4	12	1959	40	KGB
*T. marthae*	Shmidt	26.2002	80°06'N, 29°50'E		305	-	-	8	8	1986	1	KGB
*T. marthae*	SP-22	78.60	73°43'N, 161°50'W		300	-	-	-	12	1978	26	KGB
*T. marthae*	SP-22	79.69	74°25'N, 164°08'W		445	-	-	4	1	1979	10	KGB
*T. marthae*	SP-22	79.72	74°35'N, 164°00'W		795	-	-	7	1	1979	5	KGB
*T. marthae*	SP-22	79.74	74°38'N, 164°30'W		465	-	-	9	1	1979	11	KGB
*T. marthae*	SP-22	79.105	75°11'N, 170°05'W		315	-	-	27	2	1979	6	KGB
*T. marthae*	SP-22	79.108	75°13'N, 170°30'W		370	-	-	4	3	1979	4	KGB
*T. marthae*	SP-22	79.112	75°14'N, 171°10'W		455	-	-	10	3	1979	42	KGB
*T. marthae*	SP-22	79.115	75°02'N, 171°30'W		382	-	-	16	3	1979	11	KGB
*T. marthae*	SP-22	79.120	74°54'N, 171°37'W		330	-	-	24	3	1979	8	KGB
*T. marthae*	SP-22	79.122	74°55'N, 171°40'W		345	-	-	26	3	1979	5	KGB
*T. marthae*	SP-22	79.124	74°55'N, 171°55'W		355	-	-	1	4	1979	2	KGB
*T. marthae*	SP-22	79.128	74°53'N, 172°15'W		332	-	-	10	4	1979	2	KGB
*T. marthae*	Tunetz	105.6	68°00'N, 10°00'E		970	-0.96	34.9	15	6	1978	75	KGB
*T. marthae*	Tunetz	105.16	72°50'N, 14°00'E		960	-0.96	34.9	30	6	1978	1	KGB
*T. marthae*	Tunetz	105.21	74°30'N, 15°55'E		930	-0.3	34.9	6	7	1978	4	KGB
*T. marthae*	Vichegda	2.22	72°47'N, 58°51'E		380	-1.84	34.9	12	9	1975	12	KGB
*T. marthae*	Vichegda	2.30	72°00'N, 57°00'E		330	-1.8	34.8	16	9	1975	10	KGB
*T. marthae*	n/a	1/33266	79°08'N, 78°10'E		95	-	-	18	8	1930	7	ZIN
*T. parapari*	n/a	16.01/5689	Roquetas de Mar Almería, Andalucía, Spain		2	-	-	-	3	1986	31	MNCN
*T. parapari*	n/a	16.01/5700			2	-	-	-	7	1986	37	MNCN
*T. parapari*	n/a	16.01/5704	Cala Uruguay, Almería, Andalucía, Spain		15	-	-	-	7	1986	25	MNCN
*T. parapari*	n/a	16.01/5706	Playa de los Genoveses, cabo de Gata, Almería, Andalucía, Spain		2	-	-	26	3	1986	57	MNCN
*T. parapari*	n/a	16.01/5709	Nerja, Málaga, Andalucía, Spain		-	-	-	19	1	1983	1	MNCN
*T. parapari*	n/a	16.01/5711			-	-	-	28	12	1982	1	MNCN
*T. parapari*	n/a	16.01/5712			-	-	-	29	12	1983	1	MNCN
*T. parapari*	n/a	16.01/5713			-	-	-	14	6	1983	1	MNCN
*T. parapari*	n/a	16.01/5714			-	-	-	27	10	1983	3	MNCN
*T. parapari*	n/a	16.01/5716	Los Escullos, Almería, Andalucía, Spain		-	-	-	-	10	1984	9	MNCN
*T. parapari*	n/a	16.01/5717			-	-	-	-	10	1983	11	MNCN
*T. triserialis*	n/a	2508	39°48'N, 0°11'30"E		-	-	-	29	04	1996	1	MNCN

Photographs were produced at the PP Shirshov Institute of Oceanology, at the Russian Academy of Science, Moscow using a Leica DFC490 camera mounted on either a Leica M165C stereomicroscope or a Leica DMI 4000B compound microscope; at the Department of Invertebrate Zoology, Biological Faculty, Moscow State University using a Leica DFC425C camera mounted on a Leica DMI 5000B compound microscope; and at the MNCN by a Leica DFC550 camera mounted on a Leica MZ16A stereomicroscope. In order to increase contrast, specimens were stained with methylene blue (water solution).

**Figure 2.**
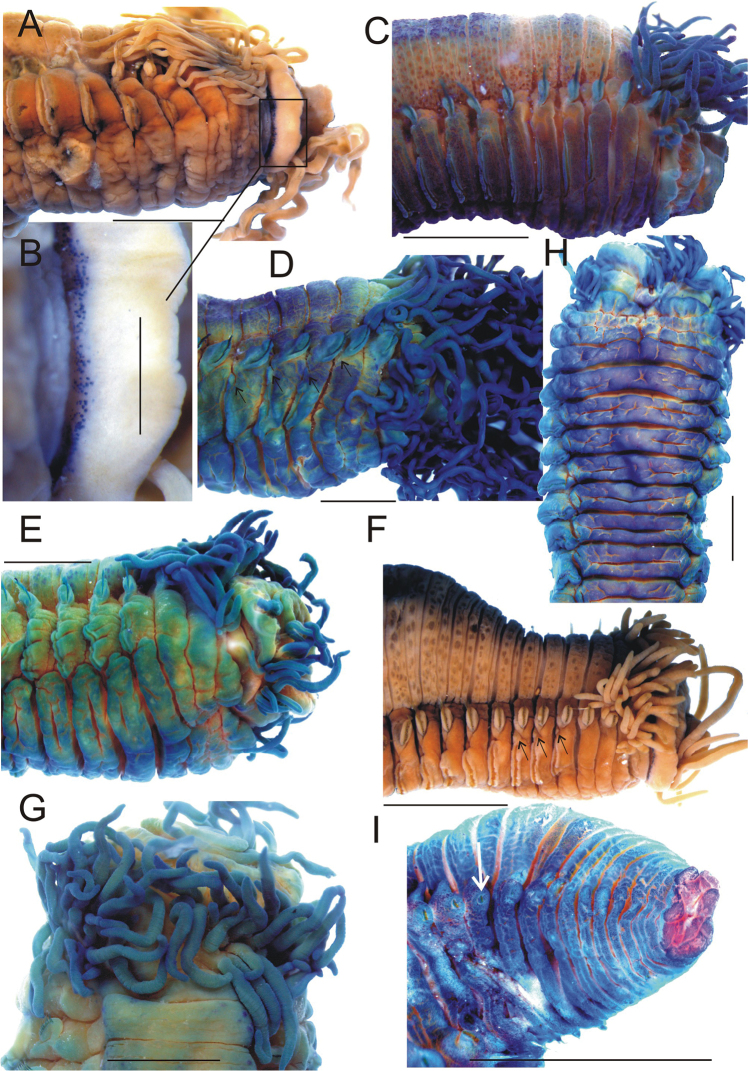
*Thelepus cincinnatus* external morphology**. A, C–I** lateral view of anterior end **B** detail, showing eyespots **G** dorsal view of anterior end **H** ventral view of anterior end **I** lateral view of posterior end (arrow indicates last segment with notochaetae). **A, B, F, I** Alaid st. 30.13 **C** Alaid st. 8 **D, E, H, G** Alaid st. 6. All worms except **A, B, F** stained with methylene blue **D, F** arrow indicates nephridial papillae. Scale bars: 2 mm except **B** 0.5 mm.

Some external morphological characters are not always and/or easily visible. Eyespots are located on back of upper lip, which is usually curved backward, so it is necessary to unbend the lip forwards to observe this feature (Fig. 2C–F). Additionally, sometimes eyespots do not form an entire band; instead, a dorsally interrupted band is present, so careful examination is required. Nephridial papillae are often poorly visible, so it is necessary to investigate several specimens, preferably well preserved and mature to get a clear picture. Counting the number of branchial filaments requires careful examination as it will appear that more rows are present due to the presence of numerous obscuring filaments. Some characters develop ontogenetically. Unfortunately usually specimens are incomplete, so it is not possible to assess age of specimen using its length. Further, length greatly depends on the degree of retraction of the worm during fixation. I recommend using relative size (assessed by eye), as length depends on muscle retraction during fixation and furthermore worms often are incomplete posteriorly. Maximum size of the largest specimen was estimated for each sample or set of nearby samples as maximum size varies between distant samples, over a species range.

## Systematics

### Terebellidae Johnston, 1846

Read and Fauchald (2018) is followed in using the family rank Terebellidae rather than Thelepodidae.

#### Thelepodinae Hessle, 1917

##### Thelepus

Taxon classificationAnimaliaTerebellidaTerebellidae

Leuckart, 1849

###### Type species.

*Amphitrite cincinnata* Fabricius, 1780.

###### Diagnosis.

Branchiae formed of numerous simple filaments arranged in more or less distinct parallel transverse rows arising from S2–S4; notochaetae from S3 (= BS2), uncini from C3 (= S5); lateral lobes absent.

###### Remarks.

The genus includes 48 species (Hsueh and Li 2016), distributed from the Arctic to the Antarctic and from the littoral to abyssal zones. The most important taxonomic characters used for species separation based on Day (1955), Hutchings and Glasby (1987), and this study are:

###### The number of branchial segments.

The number of BS varies from zero to three; most species have three BS. Only six species currently accepted as valid have two BS. Very little variation in the number of BS was observed; only one specimen amongst more than a thousand of all four species had a third branchia, on one side only. Of course, juveniles may have fewer BS, and some of the very small worms in the examined material had only one BS, or branchiae were absent. The final number of BS seems to appear when the size of the worm is approximately 1% of maximum.

###### The branchial fields from which the filaments arise.

A distinct median gap and lateral extension of the filaments appears to be constant within a species, but in species with numerous filaments both tend to change with size: as the gap becomes narrower, the extension goes further laterally.

###### The number of branchial filaments.

Some species have very few filaments in total, while others have many (10–40 or more). The number of filaments tends to increase with increasing size of the animal. Once adulthood is achieved, there is little variation in the number of filaments, independent of the size of the worms. According to our data, the maximum size of worms varies between localities for the same species, but the maximum number of filaments is relatively constant within a species. Hutchings and Glasby (1987) suggested that the relative number of branchial filaments between BS2, BS3, and BS4 is more important than the actual number of filaments. However, if there are only a few filaments, variation in their number leads to significant changes in the relative number of branchial filaments and this feature becomes unreliable.

###### The number of segments with notopodia and notochaetae.

There are two groups of species within *Thelepus*: (1) notochaetae present only on the anterior half of the body; there are numerous fully-developed segments without notopodia that differ from notopodial segments only by the absence of notopodia, and (2) species with notochaetae present for most of the body, absent only in the segments clustered near the pygidium. This difference seems to be diagnostic.

###### The number of rows of uncini.

Uncini can be in a single row or form a loop; all of the species investigated have a single row, but *T. nucleolata* Claparède, 1870, described from the Mediterranean (Gulf of Naples) has uncini forming a loop after S14. The species is poorly known and has not been recorded since the original description. The presence or absence of the loop seems to have high taxonomic value.

###### The shape of the uncinus.

The most important features seem to be the shape of the prow, the position of the attachment button, and the arrangement of teeth above the main fang-forming crest. The last character is better seen in SEM photographs, whilst the first two are better observed using a compound microscope. Three of the four investigated species with two BS have very similar U1 uncini, but other species inhabiting European waters, *T. setosus* (Quatrefages, 1866) and *T. triserialis* (Grube, 1855), have very different uncini (Fig. 1B, C). The shape of the uncini may vary along the body; they usually decrease in size but, in *T. parapari* sp. n., the shape also changes. Therefore it is best to examine and compare uncini from a specified unciniger, such as U1; comparison of previously described uncini without detail of the segment of origin has limited value.

###### Presence/absence of eyespots.

Hutchings and Glasby (1987) reported that, in some specimens of *T. plagiostoma* Schmarda, 1861 and *T. robustus* (Grube, 1878), eyespots may be absent. The species examined for this paper either have eyespots or not. Eyespots are sub-epithelial and disappear if the epithelium is macerated due to poor fixation.

###### Comparative size of notopodia.

In some species, the first notopodia are distinctly underdeveloped (for example Fig. 4D), whilst other species have all anterior notopodia of almost equal size. However, this difference may only be apparent in large worms.

###### Notochaetae.

The notochaetae of the four investigated species look very similar. The shape of the notochaetae is of limited taxonomic value, at least for the species examined here.

###### Tubes.

The tubes of all the investigated species are constructed using local material (shell fragments, small stones, spicules etc.) without specificity. Tubes are also attached to larger substrata, usually stones, if possible. Some tubes have a branched crown very similar to that reported for *Axionice conchilega* (Pallas, 1766) by Holthe (1986); this was observed in material examined in this study from the Norwegian Sea.

##### Key to European *Thelepus*

**Table:** 

1	Two BS	**2**
–	Three BS	**6**
2	Uncini in a single row throughout	**3**
–	Uncini after S14 form loop	***T. nucleolata* (Claparède, 1870)**
3	Notopodial segments present on 50–66% of body length	***T. davehalli* sp. n.**
–	Notopodial segments present on at least 90% of body length	**4**
4	Uncini of TU1 with one tooth above main fang	**5**
–	Uncini of TU1 with two teeth above main fang	***T. parapari* sp. n.**
5	Eyespots numerous (may disappear if epithelium is macerated due to poor fixation)	***T. cincinnatus* (Fabricius, 1780)**
–	Eyespots absent	***T. marthae* sp. n.**
6	Prow of uncinus well developed with a button above (Fig. 1B). Few branchial filaments	***T. triserialis* (Grube, 1855)**
–	Prow of uncinus poorly developed (Fig. 1C). Numerous branchial filaments	***T. setosus* (Quatrefages, 1866)**

##### Taxonomic remarks on European species

Species identification is straightforward when examining a series of well preserved, complete specimens. However, single and incomplete specimens (posterior absent) are often encountered. For such specimens, the researcher should initially examine the presence/absence of eyespots and then the sample locality/habitat. This information is usually sufficient for precise identification for comparatively well preserved (fresh) material. A synopsis for all known species of *Thelepus* with two branchiferous segments is given in Table 2.

**Table 2.** Synoptic character data for all known species of the genus *Thelepus* with two branchiferous segments. Abbreviations: n.d. – absence of data.

Species	eyes-pots	Filaments		number of		% body length with notopodia	loop	type locality	source
		BS1	BS2	segments	pairs of notopodia				
*T. antarcticus* Kinberg, 1866	yes	15	12	ca.100	ca.100	ca.100%	no	Antarctica	Benham (1921); present study
*T. cincinnatus* (Fabricius, 1780)	yes	<30	<22	ca.100	70–106	ca.100%	no	West Greenland	Pettibone 1954; present study
*T. crassibranchiatus* Treadwell, 1901	yes	4	2	n.d.	>38	n.d.	n.d.	Puerto Rico	Treadwell (1901); Londoño-Mesa (2009)
*T. davehalli* sp. n.	no	<20	<10	ca. 100	30–40	1/2–2/3	no	N-E Atlantic shelf	present study
*T. hamatus* Moore, 1905	yes	5	5	60	32	50%	?	Pacific Alaska	Hilbig (2000)
*T. marthae* sp. n.	no	<10	<5	ca.100	<65	90%	no	deep Arctic ocean	present study
*T. nucleolata* (Claparède, 1870)	yes	6	4	n.d.	n.d.	n.d.	yes	Gulf of Naples, Italy	Claparède (1870)
*T. pascua* (Fauchald, 1977)	no	1	1	n.d.	>=32	n.d.	no	Atlantic Panama	Fauchald (1977); Londoño-Mesa (2009)
*T. parapari* sp. n.	yes	<11	<8	ca. 70	<56	95%	no	Mediterranean	present study

###### Thelepus cincinnatus

Taxon classificationAnimaliaTerebellidaTerebellidae

(Fabricius, 1780), s. str.

**Figure 3.**
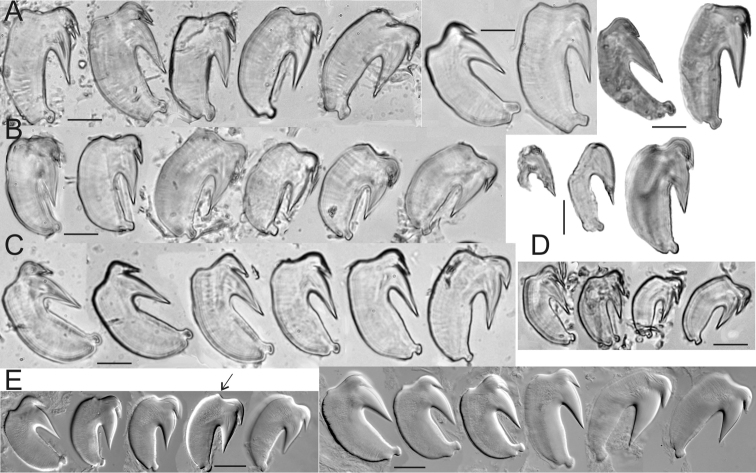
*Thelepus cincinnatus* and *Thelepus antarcticus* uncini. **A** Alaid 30.6 **B** Alaid 30.8 **C, D** Alaid 30.13 **A–C** uncini from U1 **D** uncini from posterior body **E** *Thelepus antarcticus* ZIN IV.1.2, arrow indicating hump, which is different from that in *T. cincinnatus*. Each block from one specimen, all uncini from TU1. Third block of **A** and second block of **B** shows stage of development of uncini. Scale bars: 20 μm.

Figs 2 , 3 , 11 , 12

Thelepus cincinnatus : type locality Greenland (probably Frederikshâb), type material probably never designated (Holthe 1986): ? Fauvel 1927: 271–272, fig. 95 i–m; Pettibone 1954: 327–328, fig. 37e, f; Zatsepin 1948: 154, table XXXVIII, 7 (partim); ?Hartmann-Schröder 1996: 528–530, Abb. 258; Holthe 1986: 140–142, fig. 63, map 62 (partim); Jirkov 2001: 526–527 (partim).

####### Material

(Table 1): 413 specimens from 33 stations collected at 8–1350 m, bottom temperature -1.53–7.37 °C. Ten specimens from Alaid station 6 deposited at MNCN: 16.01/17777.

####### Additional material.

*Thelepus antarcticus* ZIN IV.1.2 (5 specimens)

####### Description.

Largest specimen 140 mm in length and 5 mm in width, although some fragments distinctly larger (up to 7 mm width); maximum size estimated at over 200 mm; larger specimens had been collected at shallow depths, less than 100 m. Number of segments increased with body size; number in investigated specimens: 113.

Buccal tentacles numerous, equal to body length, grooved. Eyespots rounded subepithelial spots, black or dark brown, numerous, usually in several transverse rows on back of upper lip (Fig. 2A, B). Even smallest specimens (<0.5 mm width R/V Sevastopol st. 1769) with numerous eyespots. Specimens from deepest sample (R/V Sevastopol, st. 1580, 1350 m) also with numerous eyespots.

Branchial filaments numerous, long and tangled (Fig. 2A, C–G). Due to tangling it was impossible to count number of branchial filaments in large worms (>5–6 mm width) without removing them one by one. Maximum number of BS1 filaments ca. 20–30, extending laterally to a point level with midpoint or lower edge of row of U1 uncini; outermost filaments usually 2–3 times shorter than those most developed. BS2 with a maximum of 15–20 filaments. One specimen (from Alaid 30.13) had four filaments on BS3 on right hand side of body; length of these was equal to notopodia of same segment. Filaments attached to a transverse elevated stump in 1–2 irregular rows depending on number of filaments. Number of filaments increases with body size; small worms (1–2 mm width) have fewer than 10 filaments on BS1. Smallest specimen (Sevastopol 1769, width <0.5 mm) with no filaments. Extension of filaments laterally depends upon worm size, with filaments extending only to level of upper margin of uncinal row in small worms. Wide medial gap separating left and right groups of filaments. Lateral lobes absent. Dorsum with warts or subepithelial honeycomb, forming more or less regular rows (Fig. 2C, F); number of rows increases with size of segments and worm. Segmentation distinct. Ventrum glandular, more so with increased “wrinkling” (Fig. 2H). Poorly visible, small nephridial papillae on S4–S7 above neuropodia; those on S5–S7 largest and usually only ones visible (Fig. 2D, F, arrowed).

Notopodia commence from BS2, with anterior notopodia large and transverse. Notopodia raised on body surface or flattened, depending on whether fixation occurs whilst within or outside of tube. Notopodia of BS2 equal to or only slightly smaller than those most developed. Notopodia numerous and present on almost all segments except 10–20 posteriormost developing segments; in investigated material present on up to 106 segments. Last notopodia poorly developed, several times shorter than those most developed and almost without rami, with only a few notochaetae; last neuropodia also reduced (Fig. 2I). Part of worm without notopodia not exceeding 10% of whole body length. Notochaetae in few (ca.10) anterior segments in two transverse rows: posterior row with long chaetae, distal half (winged part) becomes stained with methylene blue, anterior row with short chaetae; other notopodia with a single row of notochaetae. Notochaetae with narrow brims (Fig. 11B).

Neuropodia from C3, tori increasing in size to U10, then becoming progressively shorter. Uncini in a single row with well-developed prow and crest and one tooth in profile (Fig. 3); within a neuropodium main fang develops first, crest develops later (Fig. 3D).

Pygidium with crenulated margin, without cirri or papillae.

####### Differential diagnosis.

Morphologically, *T. cincinnatus* is closest to *T. antarcticus* Kinberg, 1866. The original description of *T. antarcticus* is very brief. The most complete re-description is by Benham (1921). It looks very similar to *T. cincinnatus*; however, I do not believe that it is the same species, since direct comparison of material from the northern and southern hemispheres is necessary to find differences. For the present time it can be stated that, although both species are of equal size (up to 200 mm length and 7 mm in diameter), *T. cincinnatus* has at least twice as many branchial filaments as *T. antarcticus*. The five specimens investigated (length up to 5 cm) have no more than 15 branchial filaments on BS1, distinctly fewer eyespots and slightly different uncini, with a hump (Fig. 3E).

*Thelepus cincinnatus* differs from other new species described herein as indicated: from *T. davehalli* sp. n. by the presence of eyespots and the absence of numerous completely developed posterior segments without notopodia; from *T. marthae* sp. n. by the absence of eyespots and by the higher number of branchial filaments and segments with notopodia; and *T. parapari* sp. n. has a crest of uncini on TU1 with two rows in profile, while *T. cincinnatus* has only one. Other species of *Thelepus* with two pairs of branchiae and eyespots have at least three times fewer branchial filaments and all but *T. parapari* sp. n. have half the number of segments with notopodia (Table 2).

####### Remarks.

The investigated material included almost 2000 specimens (from more than 100 stations) from the high Arctic to the Mediterranean, from depths between 2 m and almost 2 km. The type locality of *T. cincinnatus* is outside the ranges of all investigated species, but *T. cincinnatus* s. str. investigated specimens perfectly agree with the description of topotypes (Pettibone 1954). It is supposed that Pettibone’s description is that of the true *T. cincinnatus*.

In some samples, specimens lacked eyespots; however, this is likely to be due to fading because specimens in same samples (with several specimens present) have eyespots, but they are paler, smaller and less numerous than is typical. This fading seems to depend on preservation method: all material with faded eyespots had been stored in formalin for over ten years. The age of samples does not influence fading significantly; although all specimens without eyespots were collected over 50 years ago, other specimens collected a century ago and kept in alcohol had retained eyespots. So absence of eyespots should not be considered to be a characteristic of this species.

Three subspecies (varieties according to original descriptions) of *T. cincinnatus* have been described (Bellan 2008) and, based on the discussion below, none are considered valid.

Thelepus cincinnatus var. andreanae McIntosh, 1922. McIntosh wrote “dorsal cephalic collar with eye-specks”; as all other *Thelepus* with two pairs of branchiae from the area near the type locality also lack eyespots, this name should be accepted as a junior synonym of *T. cincinnatus* s. str. as believed by Bellan (2008).

Thelepus cincinnatus var. canadensis McIntosh, 1885; has eyespots according to the original description. Type locality: 43°04'N, 64°05'W, 51 fms. Specimens collected near the type locality of this subspecies (R/V “Persey-3” see Table 1) did not show differences from other specimens, confirming Hartman’s (1959) acceptance of T. cincinnatus var. canadensis as a junior synonym of the stem subspecies.

Thelepus cincinnatus var. profundus Roule, 1896. The description is too short to be informative: ‘Un seul individu, différant du type par sa taille e plus petite, par son tube plus mince et couvert extérieurement d’un enduit peu épais formé de vase grise, et par la forme de ses plaques onciales; ces dernières sont plus étroites, et leurs trois dents plus espacées’. No figures are given so it is impossible to determine which species he was describing and as no type material was deposited in Paris (Solís-Weiss et al. 2004) this subspecies should be treated as a *nomen dubium*.

Other literature reports of *Thelepus cincinnatus* include:

Fauvel (1927) reported for *T. cincinnatus*; “nombreux points oculiformes”; however, most or all the area covered by the “Faune de France” seems to lie outside the range of *T. cincinnatus*, but includes the range of *T. parapari* sp. n. with eyespots, so he probably observed *T. parapari* sp. n.

Zatsepin (1948) and Holthe (1986); despite their descriptions agreeing well with *T. cincinnatus* s. str., they probably observed the other species described here, because these species’ ranges fall within those covered by their papers. The same is true for our papers (Jirkov 2001; Jirkov and Leontovich 2013), where we overlooked *T. marthae* sp. n., *T. davehalli* sp. n., and *T. parapari* sp. n. but, in this case, it is supported by re-investigation of the material.

Hartmann-Schröder (1996) reported eyespots for *T. cincinnatus* (Abb. 258), but her figures showed too few branchial filaments and no visible eyespots (they cannot be confirmed or observed in the figure shown). Either the specimen in the figure is too young (there is no scale) or she was studying a different species.

###### Thelepus davehalli sp. n.

Taxon classificationAnimaliaTerebellidaTerebellidae

http://zoobank.org/7F969CCC-1770-4B35-9373-2271D9876ACC

Figs 4 , 11

####### Material

(Table 1): 444 specimens from 27 stations, collected at depths from 94–495 m, 1.73 °C–11.3 °C. Holotype st. Sevastopol 2587. Material is deposited at the KGB, three paratypes from Sevastopol st.1453 are deposited at the MNCN 16.01/17772. Material from Aveiro (DBUA0000389.01) and Naples (MNCN 16.01/488) is not included in the type series as it was collected too far away from the type locality, despite seeming to be morphologically identical.

####### Description

(based on holotype and paratypes). Holotype with 97 segments, 32 segments with notopodia, 95 mm length. Paratypes up to 100 mm long and 5 mm wide; number of segments increased with body size, up to 91.

**Figure 4.**
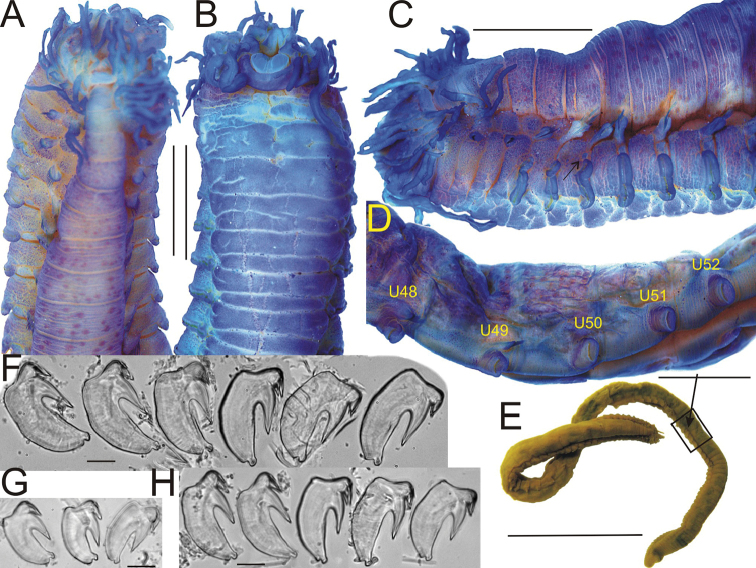
*Thelepus davehalli* sp. n. **A–C** anterior end: **A** dorsal view **B** ventral view **C** lateral view (arrowed nephridial papilla) **D** U48–U52 lateral view **E** – total view, rectangle shows position of **D** (arrow indicates last segment with notochaetae) **F–H** uncini: **F, G** U1 **H** U48, **A–F, H** Sevastopol st. 2587: **A–E** holotype **F, H** paratype **G** APEM 232335. All worms but **E** stained with methylene blue. Scale bars: 2 mm (**A–E**); 20 μm (**F–H**).

Several tens of grooved buccal tentacles as long as half body length. Eyespots absent. Branchial filaments numerous, long and tangled (Fig. 4A, C). Due to tangling, it was impossible to count number of branchial filaments in large worms (>5–6 mm width) without removing them one by one. Maximum number of BS1 filaments ca. 20 (13 in holotype), extending laterally to a point level with upper edge of row of U1 uncini. BS2 with ca. ten filaments (nine in holotype). Filaments attached to a transverse elevated stump in 1–2 irregular rows but, due to numerous filaments, there appear to be more rows. Number of filaments increases with body size; small worms (1–2 mm width) with ca. 5 filaments on BS1. Lateral extension of filaments depends upon worm size: in small worms, filaments extend only to a point level with notopodia. Lateral lobes absent. Dorsum with warts or subepithelial honeycomb, forming more or less regular rows (Fig. 4A, C); number of rows increases with size of segments and worms. Segmentation distinct. Nephridial papillae on S5–S7 above neuropodia (Fig. 4C, arrowed), usually poorly visible or not visible; papillae on S4 apparently absent. Ventrum glandular, with “wrinkling” (Fig. 4B) increasing with worm size.

Notopodia from BS2. In small worms, more or less similar, almost cylindrical; in large worms, anterior notopodia transversely flattened, those in first few anterior segments several times smaller than those that are most developed (Fig. 4). Largest specimens in each sample with about 30–40 segments with notopodia, the smallest with fewer, but even specimens ten or more times smaller than largest (by size) with over 30; the next 40–60 segments without notopodia, i.e. about 1/3–1/2 of body length without notopodial segments. Notochaetae with narrow brims (Fig. 11A).

Neuropodia from C3; tori increasing in size to U10, then becoming progressively smaller. Uncini in a single row, uncini of U1 with well-developed prow and crest with one tooth in profile (Fig. 4F, G); posterior uncini (U48) very similar (Fig. 4H).

Pygidium with crenulated margin, without cirri or papillae.

####### Differential diagnosis.

Only one previously known species, *T. pascua* (Fauchald, 1977) from the Atlantic coast of Panama, has two pairs of branchiae and no eyespots. It differs from *T. davehalli* sp. n. in its lower number of branchial filaments: single filament in BS1 and BS2 in *T. pascua*; up to 20 filaments in BS1 and up to 10 filaments in *T. davehalli*. Only one previously known species, *T. hamatus* Moore, 1905 from Pacific Alaska, has two pairs of branchiae and segments of the posterior half of the body without notopodia. It differs from *T. davehalli* in the presence of eyespots and a lower number of branchial filaments: five in BS1 and BS2 in *T. hamatus*; up to 20 filaments in BS1 and up to 10 filaments in *T. davehalli*. *Thelepus davehalli* differs from the other species described in this paper and other known species with two pairs of branchiae in the presence of fully developed segments without notopodia in the posterior 1/3–1/2 of the body.

The last biramous parapodia of *Thelepus davehalli* is well developed (not reduced), following uniramous parapodia with well-developed neuropodia, contrary to other species described in this study (Fig. 4D, E). Anterior segments lack well-developed notopodia, contrary to those in *T. cincinnatus* and *T. marthae*.

####### Remark.

Thelepus cincinnatus var. andreanae McIntosh, 1922 was described from within the range of *T. davehalli*. However, McIntosh clearly stated “Dorsal cephalic collar with eye-specks” while this new species has no eyespots.

####### Etymology.

**Figure 5.**
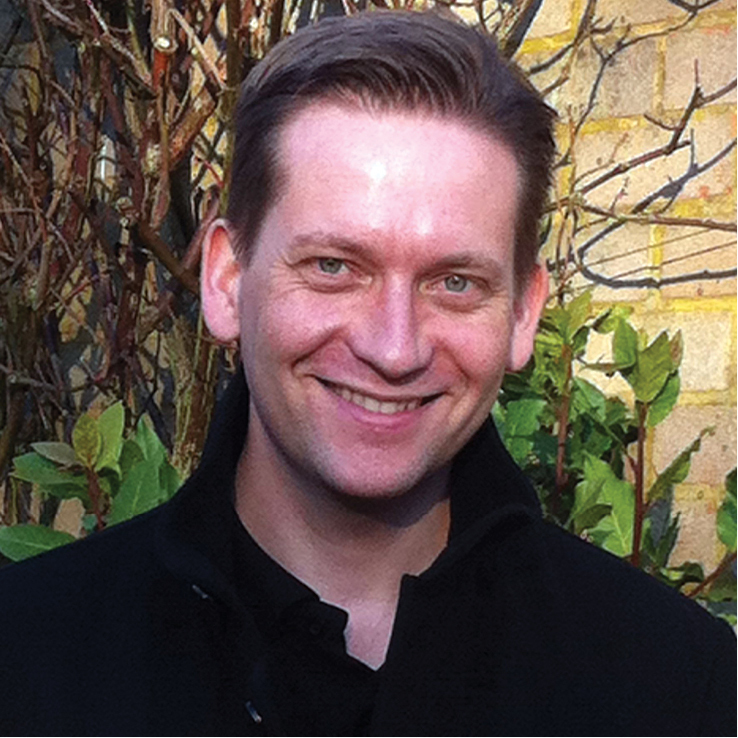
David Hall. The photograph was taken by his eldest daughter, Tara Hall.

The species is named after my friend Mr. David Hall, Head of Marine and Freshwater Laboratories, Associate Director APEM Ltd., UK (Fig. 5).

###### Thelepus marthae sp. n.

Taxon classificationAnimaliaTerebellidaTerebellidae

http://zoobank.org/10A8FCD4-3C8D-4B71-B5C6-341403C7F10E

Figs 6 , 7 , 11C

Thelepus cincinnatus : Zatsepin 1948: 154, table XXXVIII, 7 (partim); Jirkov 2001: 526–527 (partim) – non Fabricius 1780.

####### Material

(Table 1): 921 specimens from 38 stations collected from depths between 95–1,510 m, bottom temperature -1.84–2.8 °C. Holotype: R/V Tunetz cruise 105 station 6. Material is deposited at the KGB, fifteen paratypes from Alaid st. 6 are deposited at MNCN 16.01/17773, seven paratypes are deposited at ZIN 1/33266.

####### Description

(based on holotype and paratypes). Holotype with 81 segments, 55 segments with notopodia, 55 mm length. Paratypes up to 80 mm in length, 6–7 mm in width, 100 segments, last segments still in formation and clustered, not fully developed, with poorly-developed neuropodia, so not possible to count total number of segments.

**Figure 6.**
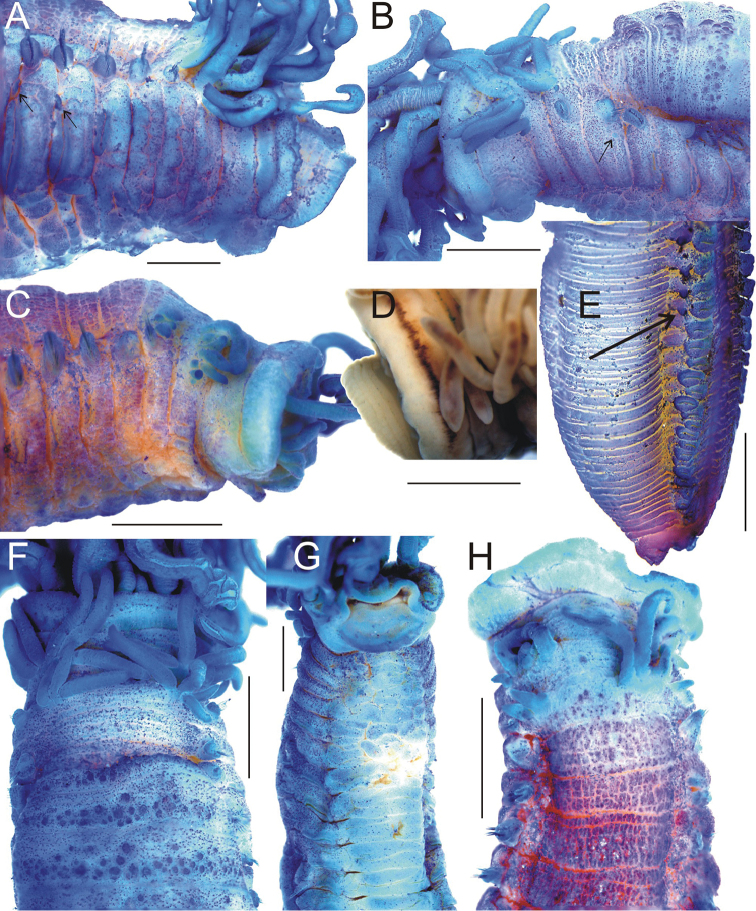
*Thelepus marthae* sp. n. external morphology. **A–C** lateral view of anterior end (arrowed nephridial papillae) **D** detail of anterior end, showing pigmented eyespots **E** lateral view of posterior end (arrowed last segment with notochaetae) **F, H** dorsal view of anterior end **G** ventral view of anterior end. **A** SP-22 st.60 **B, F, G** holotype **C, H** Alaid 30.3 **D, E** SP-22 st. 72. Scale bars: 1 mm. All worms but **D** stained with methylene blue.

Several tens of buccal tentacles, their length in fixed specimens equal to half of body length. Eyespots absent (Fig. 6A–C). BS1 with up to ten filaments (seven in holotype); BS2 with up to five (four in holotype) (Fig. 6F, H). Number of filaments increases with worm size; smallest worms, width <1 mm, with either no branchiae or with 1–2 filaments on BS1 and none on BS2. However, maximum number of filaments constant in different samples (containing sufficient worms) despite a range of maximum worm sizes across the samples. For example, largest worms from sample SP-22 st. 60 are at least three times larger than those from sample Alaid st. 3, but maximum number of filaments observed is same. Branchial filaments of BS1 extend laterally from level of notopodia of C1, to a maximum level with upper margin of uncinal row of U1. Filaments attached in a single row on an elevated stump. A wide medial gap separates left and right groups of filaments. Lateral lobes absent. Barely visible nephridial papillae on S4–S7 above neuropodia (Fig. 6A, B arrowed), in most specimens, few papillae visible, usually none. Ventrum glandular, with “wrinkling” (Fig. 4B) increasing with worm size (Fig. 6G).

Notopodia from S3, anterior notopodia almost cylindrical. Notopodia on C1, often C2, and sometimes C3 two to three times smaller than most developed notopodia (app. C10), sometimes one notopodium on C1 absent (Sevastopol 1358). Most developed notopodia transversally flattened, then reduced in size and become cylindrical again. In the most posterior segments notopodia very small; notochaetae present but several times shorter than most developed ones with no more than 10 per ramus; neuropodia also reduced to small pinnuli with few uncini. Notochaetae absent in 20–40 developing segments near pygidium (Fig. 6E); exact number difficult to determine as both annulation and neuropodia poorly developed. Some specimens also without notopodia on the 10–20 preceding reasonably well-developed segments. Number of segments with notopodia around 60 (in few complete worms available for this species), with several posterior segments lacking notopodia. However, segments without notopodia form only ca. 10% of the total worm length. Notochaetae of anterior segments two to three times longer than notochaetae of posterior segments. Notochaetae in two transverse rows: anterior row with short chaetae, distal half (winged part) becomes stained with methylene blue, posterior row with long chaetae. Notochaetae with narrow brims (Fig. 11C).

**Figure 7.**
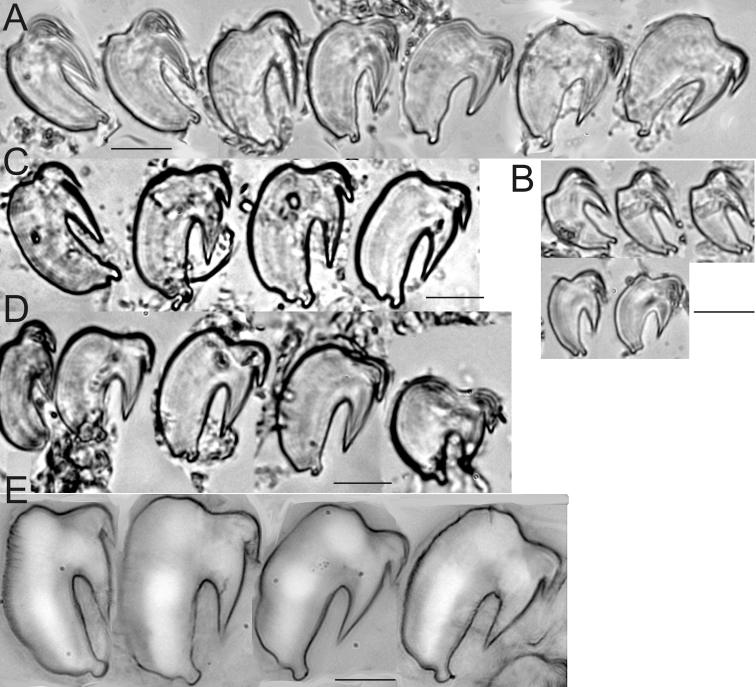
*Thelepus marthae* sp. n. uncini. **A, B** – Tunetz 105.6 **C, D** Alaid 30.3 **E** SP-22 60. **A, C–E** uncini of U1 **B** uncini of U20 from the pygidium. Each block from one specimen. Scale bars: 20 μm.

Neuropodia from C3; tori increasing in size to U10, then becoming progressively slightly shorter. Uncini in single row. Uncini of U1 with well-developed prow and crest with one tooth in profile (Fig. 7A, C–E), posterior uncini (U20 from pygidium) very similar (Fig. 7B).

Pygidium with crenulated margin without cirri or papillae (Fig. 6E).

####### Differential diagnosis.

Only one previously known species, *T. pascua* (Fauchald, 1977) from the Caribbean coast of Panama, has two pairs of branchiae and no eyespots. It differs from *T. marthae* in the lower number of branchial filaments: single filament in BS1 and BS2 in *T. pascua*; up to 20 filaments in BS1 and up to 10 filaments in *T. marthae*. *Thelepus marthae* differs from *T. davehalli* (described above) in the typically observed absence of fully developed segments without notopodia; if present, they form no more than 10% of the body length. *Thelepus marthae* differs from *T. crassibranchiatus* Treadwell, 1901, *T. hamatus* Moore, 1905 and *T. pascua* (Fauchald, 1977) (which have eyespots) in the higher number of branchial filaments and segments with notopodia. *Thelepus marthae* differs from *T. cincinnatus* and *T. antarcticus* in the lower number of branchial filaments and segments with notopodia. *Thelepus marthae* differs from *T. parapari* in the shape of its uncini.

####### Remark.

One specimen (SP-22 st. 72) has numerous spots (Fig. 6D); together forming a transverse row, as with typical eyespots but, in this case, each individual spot is longitudinal instead of rounded as in *T. cincinnatus* (Fig. 2B) and other Terebellidae. These spots are in the same place as eyespots, but their very unusual shape makes their interpretation as eyespots doubtful; other interpretations are possible.

####### Etymology.

**Figure 8.**
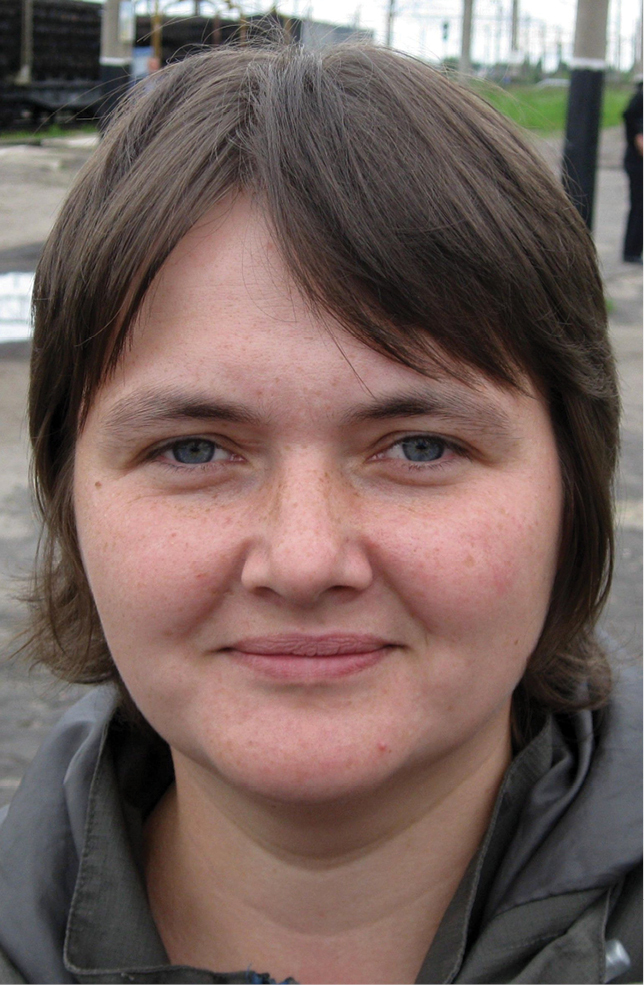
Dr. Martha K. Leontovich. The photograph was taken by the author.

Species is named after my friend Dr. Martha K. Leontovich (Fig. 8); she has described several new terebellid species.

###### Thelepus parapari sp. n.

Taxon classificationAnimaliaTerebellidaTerebellidae

http://zoobank.org/8B263E58-716A-4994-B773-E360665853B8

Figs 9 , 11D

####### Material

(Table 1): 177 specimens from 11 stations collected 26.03.1986 between rhizomes of *Posidonia*, coralligenous formations, calcareous concretions and under stones, 2–15 m, Andalusia, Spain. Holotype MNCN 16.01/17774 (previously part of MNCN 16.01/5706), 5 paratypes previously deposited in MNCN 16.01/5706 now deposited in KGB.

####### Description

(based on holotype and paratypes). Holotype with 58 segments, 50 of them with notopodia, 50 mm length. Paratypes up to 60 mm in length, 2 mm in width, 60–70 segments, posterior segments clustered and developing with poorly-developed neuropodia, so not possible to count total number of segments.

**Figure 9.**
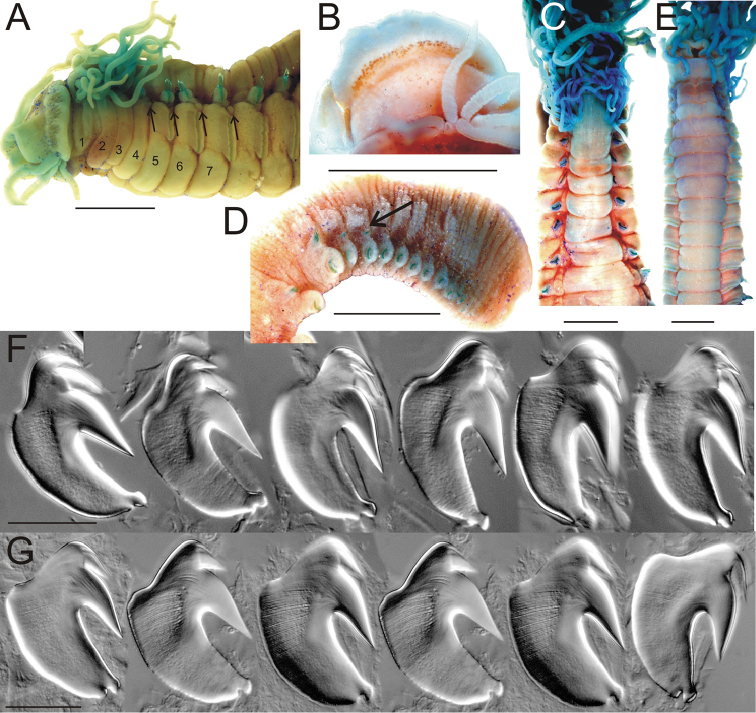
*Thelepus parapari* sp. n. **A** lateral view of anterior end (numbers of S are shown, nephridial papillae arrowed) **B** detail of anterior end, showing pigmented spots **C** dorsal view **D** view of posterior end (arrowed last segment with notochaetae) **E** ventral view **F** U1 uncini **G** U25 uncini. **A, C–E** holotype **B** MNCN 5700 **F–H** MNCN 5706. All worms stained with methylene blue. Scale bars: 1 mm (**A–E**); 20 μm (**F, G**).

Several tens of buccal tentacles, their length in fixed specimens equal to half of body. Eyespots absent in most specimens (Fig. 9A), only some with reddish eyespots forming a band without dorsal gap (Fig. 9B). Eyespots probably fade during preservation or variation in this character. Preserved body uniformly beige to yellowish, without distinct patterns of pigmentation; one specimen with eyespots with reddish spots around branchiae. BS1 with up to 12 filaments (11 in holotype); BS2 with slightly fewer filaments (eight in holotype; generally, >70% number on BS1). Filaments thin and very long, reaching more than half of corresponding segment’s width (Fig. 9A, C). Number of filaments increases as worm grows; smallest observed worms (width <0.5 mm) with 1–2 filaments on BS1 and one on BS2. Branchial filaments of BS1 attach in an irregular row on a slightly elevated stump and extend laterally to a point level with notopodia of C1 or sometimes level with upper margin of uncinal row of C3. Filaments of BS2 do not reach notopodia and usually form two rows. A wide medial gap separates left and right groups of filaments. Lateral lobes absent.

Notopodia commence from S3, almost cylindrical anteriorly; those from C1 onwards of equal size. Posterior notopodia poorly developed (almost no rami), with few notochaetae that are several times shorter than most developed notochaetae; neuropodia also reduced. Notochaetae absent only in developing segments near pygidium, approximately ten such segments, exact number difficult to determine as both annulation and neuropodia poorly developed (Fig. 9D). Characteristic number of segments with notopodia less than 60 (based on few available complete worms). Segments without notopodia from only ca. 5% of total worm length. Relatively distinct (in comparison with species described above), small nephridial papillae on S4–S7, above neuropodia (Fig. 9A). Ventrum glandular, without distinct pads (Fig. 9A, E).

Notochaetae sometimes form two distinct transverse rows: anterior row with short chaetae, posterior row with longer chaetae, distal half (winged part) becomes stained with methylene blue, but usually in one row with mixed short and long chaetae; flanges appear to be wider than in species described above (Fig. 11D).

Neuropodia from C3, tori. Uncini in a single row. Uncini of U1 with two teeth in profile above main fang, unlike three species described above (Fig. 9F). However, posteriorly, uncini have only one tooth in profile, in common with species described above (Fig. 9G).

Pygidium with crenulated margin, without cirri or papillae.

####### Differential diagnosis.

Only one previously known species, *T. pascua* (Fauchald, 1977), from the Caribbean coast of Panama has two pairs of branchiae and no eyespots. It differs from *T. parapari* in the lower number of branchial filaments: single filament in BS1 and BS2 in *T. pascua*; up to 11 filaments in BS1 and up to 8 filaments in *T. parapari*. *Thelepus parapari* differs from *T. davehalli* (described above) in the absence of fully-developed segments without notopodia. *Thelepus parapari* differs from *T. crassibranchiatus* Treadwell, 1901, *T. hamatus* Moore, 1905 and *T. pascua* (Fauchald, 1977) (all of which have eyespots) in the higher number of branchial filaments and segments with notopodia. *Thelepus parapari* differs from *T. cincinnatus* and *T. antarcticus* in the lower number of branchial filaments and segments with notopodia. *Thelepus parapari* differs from *T. cincinnatus* and *T. marthae* (described above) in the shape of the uncini of U1. *Thelepus nucleolata* (Claparède, 1870), as *Heterophenacia nucleolata*, was described from nearby (Gulf of Naples), but *T. parapari* has uncini in a single row, whilst in *T. nucleolata* they form two rows.

####### Etymology.

**Figure 10.**
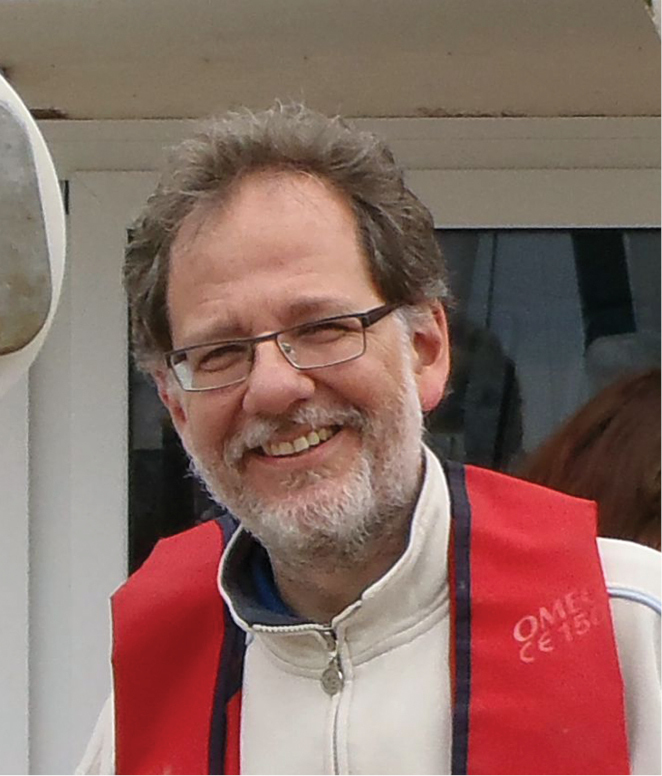
Dr. Julio Parapar. The photograph was taken by Dr. Juan Moreira.

Species is named after my friend Dr. Julio Parapar, Universidade da Coruña, Spain (Fig. 10).

**Figure 11.**
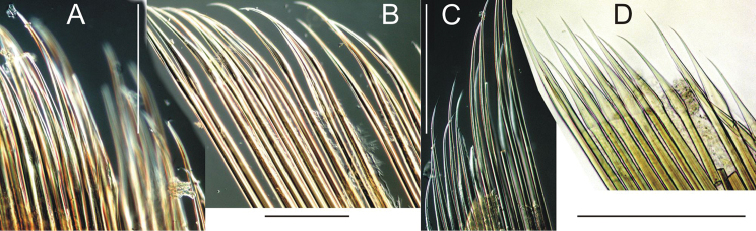
Notochaetae of *Thelepus*. **A** *T. davehalli* **B** *T. cincinnatus* **C** *T. marthae* **D** *T. parapari*. Scale bars: 0.25 mm. **A** Sevastopol 15.2587 C7 **B** Alaid 30.6 C4 **C** Tunetz 105.6 C9 **D** MNCN 5706 C10.

## Discussion of species ranges

**Figure 12.**
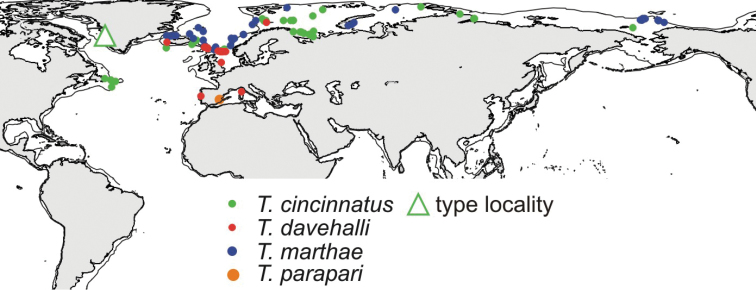
Map showing records of *Thelepus*. 500-m isobath is shown.

Species range is a good character to assist with identification. Taxonomically similar species may have different, usually complimentary, ranges and, in this instance, the number of differing ranges is few. Usually, a species’ range lies within a limited suite of ecological characters; for example, it is unlikely that the same species inhabits both intertidal and abyssal zones. On first impression, it seems that the ranges of the four species described here overlap (Fig. 12); however, in reality they are complimentary. Obviously *T. cincinnatus* s. str. is not a cosmopolitan species and it is even less widely distributed than previously supposed. Its range is limited to northern boreal and Arctic regions at least to the Chukchi Sea. In the Norwegian and Barents Seas and near Newfoundland, it was found at shelf depths from 8 to 200–400 m; in the North Atlantic south of Iceland, it occurs deeper at least up to 1300 m, so it can be expected south of Newfoundland at similar depths, in the high Arctic it is limited to shelf. I have not yet studied material from the Pacific Ocean, but as it was found in the Chukchi Sea, *T. cincinnatus* s. str. would be expected to occur in the North Pacific and it was reported by Uschakov (1955), Imajima and Hartman (1964) and Hobson and Banse (1981). Such a range (pers. obs.) is very usual in polychaetes and other benthic taxa. According to this study, at shelf depths to the south, *T. cincinnatus* is replaced by *T. davehalli* and, to the north, by *T. marthae*. *Thelepus marthae* also inhabits the Arctic slope from the Norwegian Sea to the slope of the Chukchi Sea but depth itself is not the limiting factor for the range: *T. marthae* can be found as shallow as 95 m in parts of the shelf nearby the slope. So ranges of *T. cincinnatus* and *T. marthae* are overlapping by depth limits, but not overlapping spatially. The fourth species previously identified as *T. cincinnatus*, *T. parapari*, inhabits upper sublittoral habitats in the Mediterranean (between the tidal front and the shore); in deeper water, below the tidal front, it is replaced by *T. davehalli*.

Of the other *Thelepus* species with two pairs of branchiae, *T. antarcticus* is limited to the Southern Ocean, *T. crassibranchiatus* and *T. pascua* are tropical west Atlantic species; the ranges of these species are significantly geographically removed from those of the species described here. *Thelepus nucleolata* (Claparède, 1870) is described from the shallow Mediterranean and thus is sympatric with *T. parapari. Thelepushamatus* is reported from Alaska to California (Moore 1906; Hartman 1969; Hilbig 2000) and is sympatric with *T. cincinnatus* at least in British Columbia: *T. cincinnatus* was reported from this province by Berkeley (1968) and it is the type locality of *T. hamatus*, despite not having been listed by Berkeley (1968).

## Supplementary Material

XML Treatment for Thelepus

XML Treatment for Thelepus cincinnatus

XML Treatment for Thelepus davehalli

XML Treatment for Thelepus marthae

XML Treatment for Thelepus parapari
